# Optimized ICPCVD-Based TiO_2_ for Photonics

**DOI:** 10.3390/ma15072578

**Published:** 2022-03-31

**Authors:** Aurore Andrieux, Marie-Maxime Mennemanteuil, Nicolas Geoffroy, Mélanie Emo, Laurent Markey, Kamal Hammani

**Affiliations:** 1 Laboratoire Interdisciplinaire Carnot de Bourgogne (ICB), Université Bourgogne Franche-Comté, 21078 Dijon, France; marie-maxime.gourier@u-bourgogne.fr (M.-M.M.); nicolas.geoffroy@u-bourgogne.fr (N.G.); laurent.markey@u-bourgogne.fr (L.M.); kamal.hammani@u-bourgogne.fr (K.H.); 2 Institute Jean Lamour, Université de Lorraine, 54000 Nancy, France; melanie.emo@univ-lorraine.fr

**Keywords:** titanium dioxide, optical properties, plasma-assisted CVD, photonics

## Abstract

We propose obtaining TiO_2_ films by ICPCVD for the fabrication of low-loss waveguides. The challenge is to produce a dense and homogeneous layer with a high refractive index and low absorption in the visible range. Crystallized layers with features such as grains and amorphous layers have a rather low index for the application targeted, so we aimed for an intermediate state. We investigated the influence of plasma power, pressure, deposition time and annealing temperature on the structural, crystalline, and optical properties in order to tailor them. We showed that crystallization into rutile at the nanoscale occurred during deposition and under wisely chosen conditions, we reached a refractive index of 2.5 at 630 nm without creating interfaces or inhomogeneity in the layer depth. Annealing permits one to further increase the index, up to 2.6. TEM analysis on one sample before and after annealing confirmed the nano-polycrystallization and presence of both anatase and rutile phases and we considered that this intermediate state of crystallization was the best compromise for guided optics.

## 1. Introduction

Titanium dioxide (TiO_2_) is already massively used for its photocatalytic effect or hydrophilic properties [1]. For years, in optics, it has only been used as a coupling material in thin film, but its optical properties have been neglected. We have recently shown that its relatively high refractive index (>2) and the corresponding nonlinear index allows one to generate an octave spanning supercontinuum thanks to its high Raman gain [2]. Other studies have shown that its negative thermo-optic coefficient allows for a thermal waveguide [3]. The relative ease of fabrication allows one to also dope the waveguide with rare-earth ions [4] or to target metal grating couplers into the layer [5]. TiO_2_ can be obtained by different elaboration methods such as sol–gel [6], atomic layer deposition (ALD) [7], e-beam evaporation [8], sputtering [9], and plasma-enhanced chemical vapor deposition (PE-CVD) [10]. The deposited thin film can be amorphous, anatase, rutile, even brookite, or a mix of these phases. Physical sputtering is the most common deposition technique for an optical layer, allowing amorphous or crystalline anatase TiO_2_ to be obtained. However, a columnar structure or gradients in the layer is often deplored [11,12]. It can be attributed to a temperature elevation during the deposition or to a change in the growing mechanism [13], which can be driven by stress building up inside the layer [14]. A columnar structure or any other inhomogeneity in the volume of a photonic film should be avoided because of possible optical losses by diffusion at the interface after structuring (including sidewalls roughness) and because it is concomitant with higher defect concentration, impurities, or porosity in the film [15]. Extrinsic sources of losses such as roughness of the top surface should also be considered since this roughness can be an outcome of a columnar or porous morphology. Bradley et al. showed that an amorphous film had a lower refractive index than a crystalline one but propagation losses in the crystalline anatase phase were higher due to a less homogeneous morphology [16]. Hegeman et al. recently developed low-loss TiO_2_ waveguides consisting of an amorphous phase with an anatase upper layer [17]. This paves the way to several applications of TiO_2_ films such as photodetectors [18], diffracting Bragg reflectors for light emitting diodes [19], low-index films for photonic-to-plasmonic mode interface [20], or high-index waveguides for nonlinear applications. However, for optic applications, especially photonic waveguides, process parameters should be chosen and controlled carefully in order to achieve appropriate crystallographic structure and morphology of the deposited thin films. We assumed that a good candidate for guided optics should have a high refractive index in order to obtain the best confinement and a compact morphology, allowing for a smooth sidewall after etching of the film to obtain waveguides. We developed a TiO_2_ deposition process in an inductively coupled plasma (ICP) source CVD tool to address these requirements.

## 2. Materials and Methods

In this work, we used an ICPCVD from Oxford Instruments (Yatton, Bristol, UK; PlasmaPro Cobra 100 model with a 18.4 cm wide ICP-type source) to deposit TiO_2_ onto a silicon substrate. A titanium isopropoxide (TTIP) precursor (purity 95% from Strem Chemicals, Newburyport, MA, USA) was heated in a stainless-steel pot up to 70 °C to reach the vaporization transition and 50 sccm of argon was bubbled through it to obtain a 1.8 mTorr overpressure in the chamber. The gas injection ring was close to the lower electrode (“table”) where the sample lay. A total of 100 sccm of O_2_ flowed from the top of the ICP tube during the deposition and fully supported the plasma without the addition of any other gas. The pressure in the chamber was regulated thanks to a throttle valve. The table temperature was set to 200 °C.

We investigated the influence of the ICP source power, the table power, and the chamber pressure effects on the TiO_2_ thin film properties. The radiofrequency (RF) power of the ICP source varied from 800 W to 1500 W, which was substantially higher than in previous studies [14,21,22,23]. Note that it is actually not straightforward to make a comparison since the source diameter and the exposed area of the lower electrode are probably not the same. The RF power applied to the table was set to 0, 50 W, or 100 W. The difference between the electrical floating potential of the ICP plasma and the potential of the lower electrode results in a bias value and the same value can be obtained for a different set of table RF power and ICP source RF power. The working chamber pressure ranked from 12 to 50 mTorr.

The film thickness, refractive index n, and absorption coefficient k were estimated by spectroscopic ellipsometry (Jobin-Yvon HORIBA, Longjumeau, France; Uvisel 1, incidence angle 70°). The stack defined in the ellipsometric model contained a single layer of TiO_2_ deposited on silicon and n and k were determined by a “new amorphous” dispersion formula derived by Horiba Jobin-Yvon based on the Forouhi–Bloomer formulation. This dispersion formulation was established in order to give a Lorentzian shape to the expressions of the extinction coefficient and refractive index. The refractive index and absorption coefficient can be written through these equations:

(1) $$n(\omega )=n_{\infty }+\frac{\frac{f_{j}}{\Gamma_{j}}\left(\Gamma_{j}^{2}-{\left(\omega_{j}-\omega_{g}\right)}^{2}\right)\left(\omega -\omega_{j}\right)+2f_{j}\Gamma_{j}\left(\omega_{j}-\omega_{g}\right)}{{\left(\omega -\omega_{j}\right)}^{2}+\Gamma_{j}^{2}}$$

(2) $$k(\omega )=\{\begin{array}{c} \frac{f_{j}{\left(\omega -\omega_{g}\right)}^{2}}{{\left(\omega -\omega_{j}\right)}^{2}+\Gamma_{j}^{2}}\;\mathrm{for}\;\omega >\omega_{g}\\ 0\;\;\;\;\;\;\;\;\;\;\;\;\;\;\;\;\;\mathrm{for}\;\omega \leq \omega_{g} \end{array}$$

where *f_j_* is related to the amplitude of the extinction coefficient peak; Γ*_j_* is the broadening term of the absorption peak; *ω_j_* is approximately the energy at which the extinction is maximum; and *ω_g_* is the energy band gap. The set of lowest chi-squares (χ²) obtained for the fittings ranged from 1.7 to 16.7, depending on the samples, which is a sign of good fitting quality.

The morphology of TiO_2_ layers was observed using a scanning electron microscope (SEM-JEOL, Tokyo, Japan, model 6500, SE detector) on cross-section after cleaving of the sample. The crystalline structure was investigated by X-ray diffraction (XRD-Bruker, Karlsruhe, Germany; D8-A25, Cu source, LynxEye XE detector) and complemented with transmission electron microscopy (TEM) observations (JEOL, Tokyo, Japan; JEM ARM-200F Cold FEG, 200 kV) in the second part of the paper. The phases were identified with the DIFFRAC.EVA software (v5.2) and the crystallite sizes were determined by refinement of the diffractogram with the Topas software (v6, HKL phase type–Pawley method). Chemical surface composition verification was performed with the help of X-ray photoelectron spectrometry (XPS-ULVAC-PHI, Tokyo, Japan; VersaProbe, Al Kα1). We focused the study on O 1s and Ti 2p levels in order to calculate the atomic concentrations and to determine the chemical environment on each element. Multipak (ULVAC-PHI^TM^ v9.0) was used for quantification and other data treatments were carried out using Casa XPS software (version 2324PR1-0). Adventitious carbon (C1 s level at 284.8 eV) was used for energy calibration.

## 3. Results and Discussion

### 3.1. Parametric Study Using ICPCVD Tool

#### 3.1.1. Results

The TiO_2_ layer thicknesses were measured between 215 nm and 385 nm, depending on the deposition conditions used. Table 1 presents the comments on the morphology and crystallographic structure along with the index, the deposition parameters and the resulting bias values on the table during deposition. 

**Figure 1:**
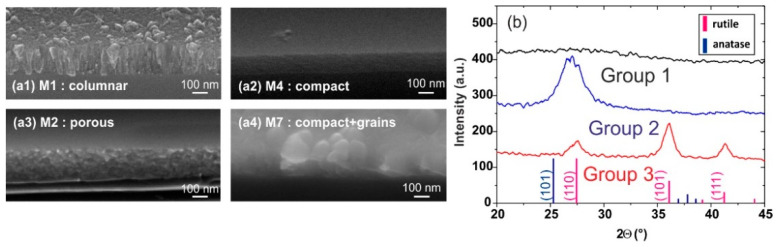
(**a**) SEM images and (**b**) XRD patterns obtained on TiO_2_ layers, illustrating the typical morphology and crystalline structure detailed for each sample in Table 1.

**Figure A1:**
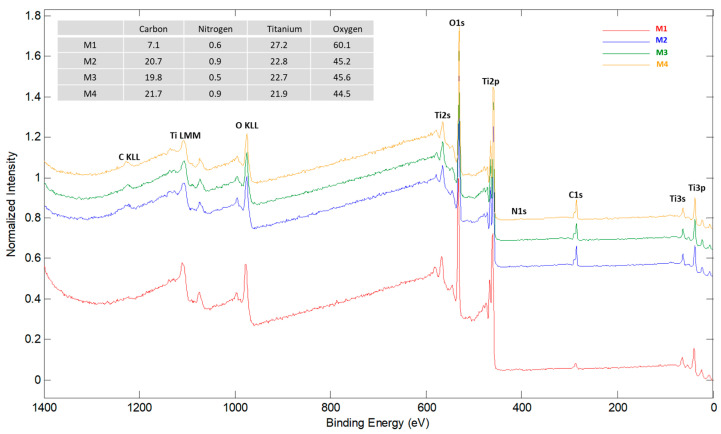
X-ray photoemission survey spectra of M1, M2, M3, and M4 layers with associated percentage atomic concentrations per element as displayed in the embedded table. Auger and XPS peak identification appear in bold black letters.

All layers had an absorption coefficient k lower than 1.10^−5^, which actually fell below the detection limit of ellipsometry estimated to be ~1.10^−3^ and the index ranged between 2 and 2.33. This is consistent with typical values for amorphous or poorly crystallized layers obtained by PE-CVD at this temperature, ranging from under 2.1 for Borras et al. with an electron cyclotron resonance-type source [24] up to 2.34–2.35 for Szymanowski et al. (conductively coupled plasma source) and Makaoui (RF “Helicon” source) [23,25]. Rico et al. and Nizard et al. reported a value of 2.19 (microwave and RF source, respectively) [26,27] and Sobczyk et al. also obtained 2.2 with a RF source [22]. We present the typical XPS spectra obtained on the M1, M2, M3, and M4 layers in Figure A1. According to the XPS measurements, the layers were stoichiometric, with some samples having an O/Ti signals ratio slightly exceeding 2 (±0.2) at the surface, attributed to surface contamination with carbon–oxygen compounds and OH^−^/H_2_O groups. We identified four types of morphologies: columnar, porous, compact, and compact with increased surface roughness (grains), as illustrated by the four SEM images in Figure 1a. Concerning the crystalline structure, the XRD pattern of the deposited layers can be classified into three groups, as illustrated in Figure 1b.

Group 1 consisted of peakless XRD patterns such as in samples M1 and M30, or a pattern that exhibited a slight bump centered at the 2θ-position (27.4°) such as in samples M2, M3, M4, and M8. We considered those layers as amorphous, with perhaps a crystallization onset. Group 2 was composed of samples M5, M7, and M9: their XRD pattern presented a broad peak centered at 27.4°, which might be the result of contributions from both the (110) rutile peak and (101) anatase peak. Samples M10, M11, M12, M14, and M17 XRD patterns showed secondary (101) and (111) rutile peaks in addition to the anatase or rutile peak (such as in the second group’s typical pattern) and formed group 3. The peak widths tended to indicate that layers were composed of small nanocrystallites (probably about 10 nm in diameter or less), as will be confirmed in Section 3.2.2. with the TEM investigations on a thick and annealed layer. Anatase crystallites have been found in the literature [15,27] and Makaoui showed the presence of both anatase and rutile phases by XRD in his thesis, but only anatase nanocrystallites were seen in the TEM image [25].

#### 3.1.2. Discussion

**Figure 2:**
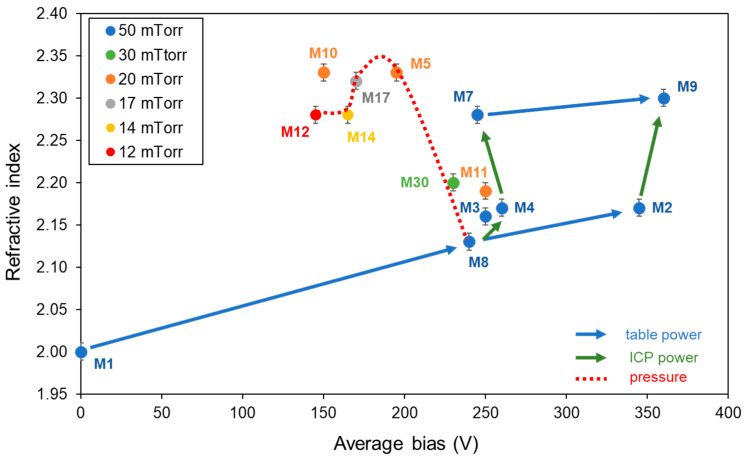
Evolution of the refractive index with the average bias during deposition for all tested conditions and illustration of the influence of an increase in each of the three parameters on the index.

Figure 2 shows the evolution of the refractive index at 630 nm as a function of the average bias value.

At a constant source power, increasing the table RF power tended to increase the refractive index, as seen with the evolution from M1 to M8 to M2 and from M7 to M9 (blue arrows). The XRD analysis of layers M1, M8, and M2 showed that the amorphous nature of the layer was preserved regardless of the table power applied for an ICP power set at 1000 W. The evolution of the index here was only due to changes in the morphology. In fact, sample M1 had the lowest index value and the layer appeared to be columnar and very porous. In this particular case where no RF power was applied to the table, we assumed that the mobility of the atoms adsorbed onto the sample surface was low due to the low energy of the incoming species (the average bias being 0 V), leading to many voids. Sample M8 had a compact morphology: the layer density might have been enhanced by the application of table power through ion bombardment on the growing film. Further increase in the bias by increasing the table power might be detrimental since the M2 layer appeared to be porous. Deposits carried out with an ICP power at 1500 W with 50 W and 100 W table power (M7 and M9 conditions), respectively, led to a similar crystalline structure of the layers with a peak position and width consistent with the presence of both anatase and rutile (group 2); further investigation into the M7 deposition conditions suggests that it is mainly rutile. The difference in the index values between M7 and M9 might be from changes in morphology or from a more advanced crystallization state, both promoted by the increase in the ion energy.

The ICP source power therefore seems to play a more critical role in increasing the refractive index. Layers obtained with a low or moderate ICP power showed a low refractive index not exceeding 2.2, corresponding to an amorphous structure. The increase in the ICP power may produce more radicals in the plasma [28], and thus provide more energy to the growing film through the ion bombardment (even if the bias values are close), promoting film densification and crystallization. At constant table power and increasing ICP source power, there was indeed an increase in the index (green arrows) from M8 to M4 to M7 (1000 W, 1200 W, and 1500 W for ICP power, respectively) as well as for M2 to M9 (1000 W and 1500 W, respectively). For the highest ICP power, grains appeared on the surface of the TiO_2_ layer (M7 and M9). In contrast, the decrease in ICP power below 1000 W led to the formation of a columnar structure (M3).

All previously mentioned samples were obtained at 50 mTorr pressure. We investigated lower pressure values at fixed ICP and table powers (1000 W and 50 W, respectively) and found that pressure was a key parameter to achieve a high index without leading to grain formation, which is undesirable for photonic applications. The red dashed line in Figure 2 represents the evolution of the index with increasing pressure (which is linearly linked to the bias value). Starting from 12 mTorr (M12), the index increased with pressure, reaching a maximum value between 17 and 20 mTorr. The accuracy of the index measurement did not allow us to clearly distinguish the best of these two pressure values (M17–M5). At 30 mTorr (M30), the index dropped abruptly from 2.3 to 2.2 and decreased again at 50 mTorr (M8). From a plasma properties perspective, at low pressure, the plasma spread more in the chamber and then became less collisional. Thus, the produced radicals lose less energy inside the plasma and their energy is more efficiently communicated to the forming layer through ion bombardment, in particular permitting the crystalline arrangement. Despite the high ion bombardment on the surface due to the low pressure, the average bias measured remained moderate (<200 V) due to the few species present in the plasma, and this led to slightly porous layers (as mentioned in Table 1). However, this morphology seems to affect the refractive index of the layers in a less preponderant way than their state of crystallization, since the index values of those layers were still around 2.3.

**Figure 3:**
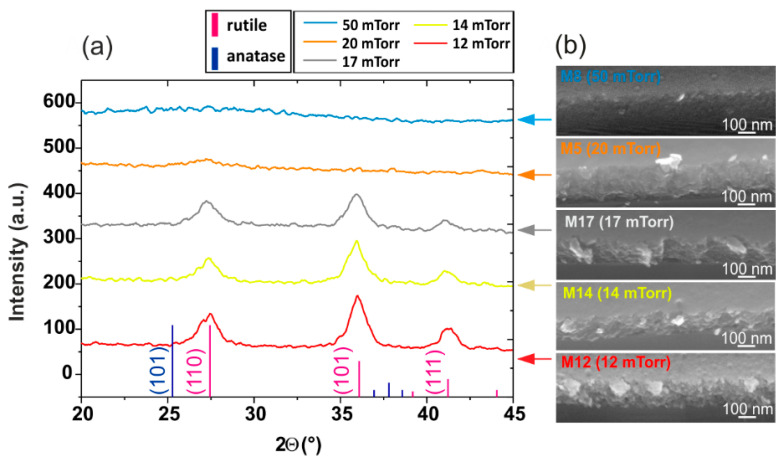
XRD pattern for four different pressures (**a**) with associated layer SEM images (**b**).

The SEM images in Figure 3b show that the sample deposited at 50 mTorr revealed a more homogeneous and compact morphology than the layers obtained at lower pressure: it confirms that the increase in pressure facilitates the layer compactness. Though the increase of the refractive index over the pressure range 12–20 mTorr can be explained by the densification of the layers, the crystallinity has to be explored to understand the drop at 30 mTorr. The XRD patterns obtained for layers deposited at 12, 14, and 17 mTorr showed a crystallization in the rutile phase with a preferential orientation along the directions (101) and (111). For pressure above 20 mTorr, the layers were considered predominantly amorphous, as seen in their XRD patterns in Figure 3a, which is consistent with the lower refractive indices obtained. While the XRD pattern appeared almost identical for the M12 and M17 layers, the change in the refractive index was not negligible and close to 0.04. The lowest index obtained at 12 mTorr (M12) may be attributed to its markedly more porous morphology (Figure 3b). Furthermore, while the XRD patterns of the M17 and M5 (deposited at 20 mTorr) were substantially different, their refractive indices were almost identical. This could suggest that the difference in compactness could compensate for a less advanced state of crystallization. In this pressure range, optimization of the refractive index can therefore be based on a compromise between the compactness of the layer obtained at higher pressure and the crystallization promoted by lower pressures.

From the trends previously discussed, we attempted to combine the best deposition conditions, meaning high ICP power (1500 W), medium table power (50 W), and medium pressure (20 mTorr). As expected, the highest index value 2.33 was reached (sample M10). It was revealed to be slightly porous and crystalline. In a surprising way, M11, deposited at higher table power (100 W) combined with a high ICP power and a medium pressure, had a lower index in spite of having a compact morphology and well-defined rutile peaks. The average bias value was 250 V (compared to 150 V for M10) and, as suggested by Makaoui [25], the decrease in refractive index with increasing bias, other conditions being the same, can be attributed to defect generation inside the layer.

For further study, we focused only on the five deposition conditions that were the most promising in terms of both refractive index (>2.28) and a rather compact morphology (M5, M14, M7, M9, and M10).

### 3.2. Thick Layer and Post-Deposition Annealing

#### 3.2.1. Results

These five conditions were reproduced with longer deposition time, thus producing thicker layers, around 550 nm, more suitable for photonic waveguide application. These new samples are similarly called with a “T” added to their name. The layer thicknesses ranged from 434 to 648 nm. An increase in the refractive index is expected with longer deposition time, caused by an increase in the density or by having a change in the crystallinity, as reported by several authors [22,25,26,29], without damaging the homogeneous morphology. A figure of merit S is defined as the percentage increase in index between the thin and the thicker layer normalized by its percentage increase in thickness. Refractive indices and thicknesses of the as-deposited thin and thick layers are presented in Table 2, along with the S value. Again, all layers had an absorption coefficient k lower than 1.10^−5^ (below the detection limit) and are not specified in Table 2.

**Figure 4:**
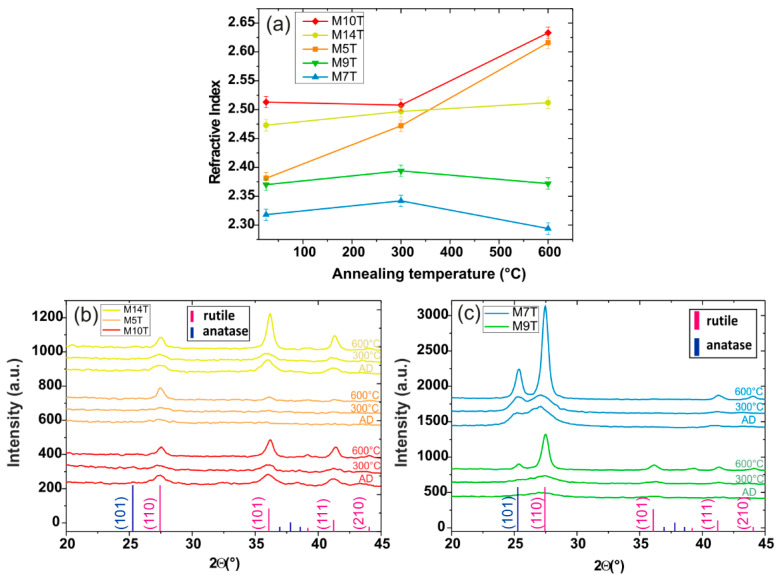
(**a**) Evolution of the films’ refractive index with the annealing temperature. (**b**,**c**) XRD patterns for the as-deposited (AD) and annealed samples with thick TiO_2_ films.

Depositions were followed by annealing for 2 h at 300 °C and 600 °C in a protective argon atmosphere. Refractive index evolution with the annealing temperature and all XRD patterns for the as-deposited and annealed samples are presented in Figure 4.

#### 3.2.2. Discussion

S values allow us to partially remove the interdependency between the thickness and the index when comparing the index evolution with the different conditions. Indeed, with the highest S observed, sample M9 stood out. Its index evolution was rather different from other deposition conditions. While the increase in thickness between the two samples was only 50 nm, the index rose by 0.7, giving a figure of merit value more than a decade higher than other samples. The SEM observation indicated that the M9T sample had a larger surface grain size for the thick layer compared to the M9 thin layer (not shown here). Regarding the ellipsometry measurements, a top layer simulating surface roughness, composed of 7.4% of air in the TiO_2_ layer with 24 nm thickness had to be added to the model to consider this surface change and obtain a good fit of the experimental data with the model. This surface feature and the increase in the index can be correlated with the XRD pattern of M9T (presented in Figure 4b), showing the appearance of secondary rutile peaks.

**Figure 5:**
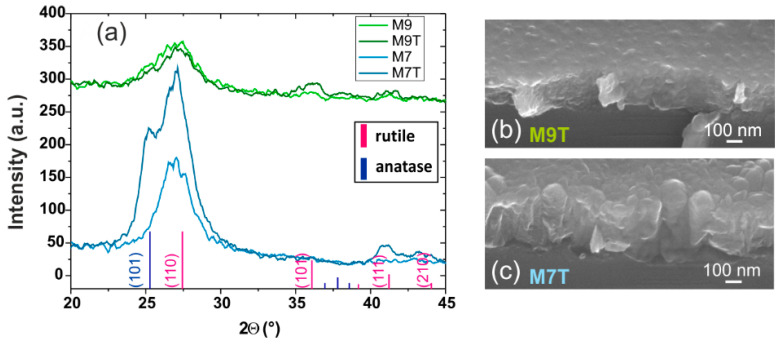
(**a**) XRD pattern evolution with film thickness for the M9 and M7 conditions and corresponding layer SEM images (**b**,**c**), respectively.

The M7T pattern showed the appearance of the (111) rutile peak at 41.4° and the broad peak observed on the analysis of the thin M7 layer was widened, clearly showing the growth of the anatase phase with the deposition time. However, the refractive index increased only slightly, with a S value being in the range of 0.030. This may be the result of an important grain growth inside the M7T layer, observed on the surface by SEM in Figure 5c (the percentage of air obtained after the fitting procedure in ellipsometry for the rough top layer was 30.5% over 20 nm for M7T), negatively affecting the refractive index, perhaps through the creation of interfaces.

M7T and M9T indices are considered as stable with annealing temperature (Figure 4a) and their XRD patterns showed a better crystallinity of the structure with a clear peak narrowing at 600 °C (Figure 4b). The decomposition of the main peak for M9T at 600 °C indicates the presence of the anatase phase. Crystallites size were a few nanometers in diameter for rutile and about 10 nm for anatase up to 300 °C in both cases and increased to ~25 nm at 600 °C for both anatase and rutile. As observed for the comparison of thin and thick layers, the refractive index stagnated despite the strong crystalline growth observed in the XRD patterns due to the presence of grains in layers M7T and M9T.

As the XRD patterns of M5T, M10T, and M14T as-deposited were similar to those of the thin layers (not presented here), it is therefore unclear whether the increase in index can be attributed first to the growth of nanocrystallites or to a higher density. 

The evolution of the refractive index for M14 with the annealing temperature was low, rising from 2.47 to 2.51: the XRD pattern stayed only as rutile, with narrowed peaks at 600 °C indicating a better crystallinity (Figure 4c). The largest increase was obtained for M5T with an index value of 2.38 for the as-deposited layer, which increased up to 2.61 after the annealing at 600 °C. While the as-deposited M5T patterns showed only one small and broad peak centered around the main rutile peak (25.4°), the annealing at 600 °C revealed three new broad rutile peaks corresponding to the secondary orientations (101), (111), and (210) at 36.1°, 41.2°, and 44°, respectively. No significant change was observed on the index of M10T after annealing at 300 °C, but annealing at 600 °C led to an index close to 2.6. The crystallite sizes were less than 10 nm for the samples as-deposited and after annealing at 300 °C and increased to be a little higher than 25 nm for the samples annealed at 600 °C. The evolutions of M14T, M5T, and M10T were consistent with starting crystallization during deposition, which continued during annealing with the growth of the crystallites, while the temperature also adds supplementary energy to reorganize and compact the layer.

**Figure 6:**
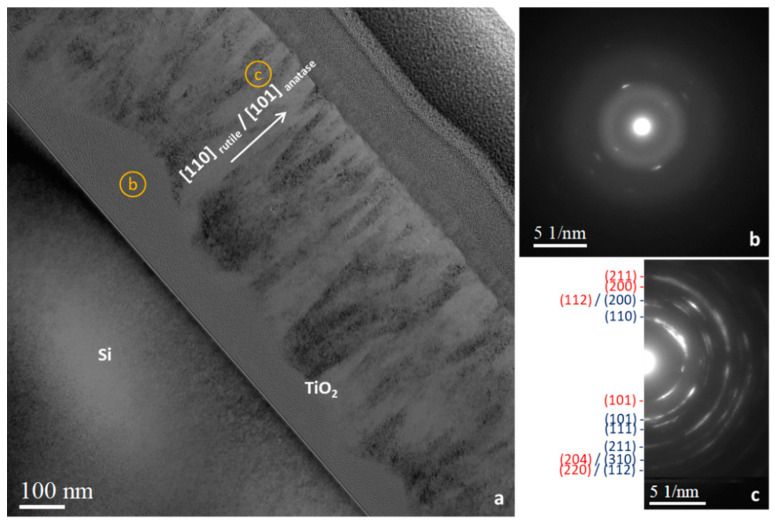
(**a**) TEM image of as-deposited TiO_2_ thin film (M7T) with corresponding selected area electron diffraction patterns near the Si/TiO_2_ interface (**b**) and at the TiO_2_ film surface (**c**). The diffraction rings have been labeled with the corresponding lattice planes (hkl) (anatase in red, rutile in dark blue).

To confirm and better understand the unexpected presence of the anatase phase despite having conditions permitting the rutile phase to grow, a cross-section sample from the M7T layer (most crystallized sample) was prepared by the FIB lift-out technique: from the TEM image (Figure 6a), two domains were highlighted, one near the Si/TiO_2_ interface, which was hardly crystallized (Figure 6a,b), and one larger part of the layer (thickness of 400–450 nm), which was polycrystalline (Figure 6a,c). As seen in the selected area electron diffraction (SAED) pattern in Figure 6c, this polycrystalline domain can be attributed to a mixture of rutile (d110 = 3.3 Å) and anatase (d101 = 3.5 Å) phases.

**Figure 7:**
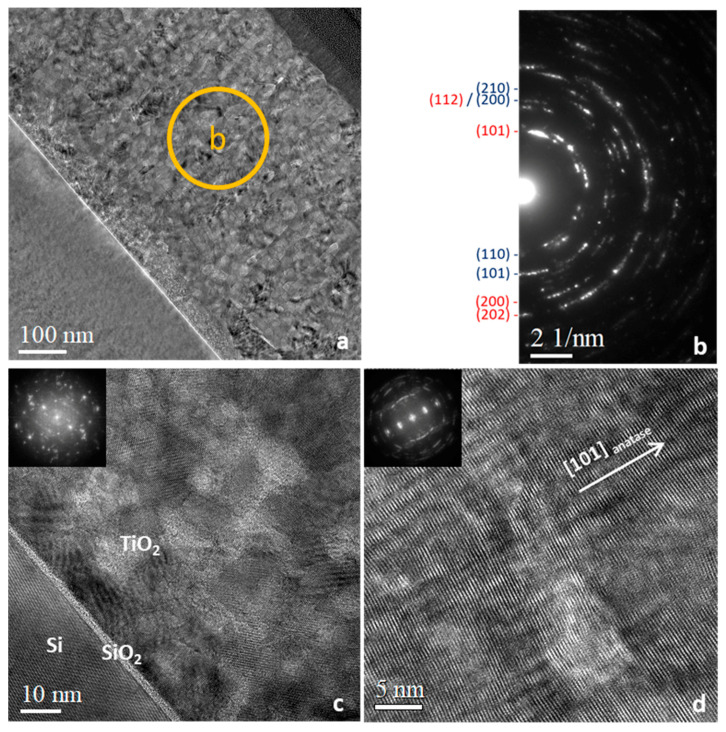
TEM images obtained on the 600 °C-annealed TiO_2_ thin film (M7T) (**a**) and corresponding selected area electron diffraction (**b**), HRTEM micrographs near the Si/TiO_2_ interface (with visible native SiO_2_) (**c**) and at the TiO_2_ film surface (**d**). The diffraction rings have been labeled with the corresponding lattice planes (hkl) (anatase in red, rutile in dark blue) on (**b**).

TEM observations were also performed on the annealed M7T sample to observe the stability of the structure: contrary to the M7T sample, two highly-crystallized domains were observed (Figure 7a). The spot-rich electron diffraction pattern (Figure 7b) indicates that the upper layer was mainly composed of a mixture of rutile (d110 = 3.3 Å) and anatase (d101 = 3.5 Å). Only a few parts all over the sample remained rutile only (not illustrated here). The HRTEM image of the upper phase showed that anatase had a preferential orientation along the (101) direction on a few nanometer range (Figure 7d), while the phase near the TiO_2_/Si interface was polycrystalline with no preferential orientation (Figure 7c). The polycrystalline state was clear in these images due to the Moiré effects reflecting the presence of several orientations along the width of the sample relative to the electron beam. We can understand the formation of anatase during annealing as the crystallization of small amorphous domains in-between existing rutile domains. In fact, it has been shown theoretically that the thermodynamic stability between anatase and rutile changes depending on crystallite sizes through promoting the anatase phase for small crystallite sizes [30].

We can assume that most of the as-deposited layers (thin or thick) in this study were composed mainly of nano-polycrystalline phases, even if their XRD patterns did not present well defined peaks. The ICPCVD environment promotes early stage crystallization in the growing film that can carry on if the deposition time is long enough and/or after annealing. We cannot see clear interfaces between the crystallites themselves nor the domains having a similar orientation, which is encouraging for the low optical propagation loss objective.

## 4. General Discussion and Perspectives

The previous sections have shown the ability to produce thin film layers with indices ranging from 2.0 to 2.6. However, for a targeted application such as guided optics, all samples cannot be used. Layers that appear amorphous had an index of less than 2.2. This value is quite low to consider highly confined mode propagation in the waveguides that might be fabricated based on those layers. We confirmed by XRD and TEM investigations that the ICPCVD-based layers could be considered as amorphous for thin layers only in some specific deposition conditions (group 1) but for the second and third groups of samples, the crystallographic structure must be considered as nano-polycrystalline, with either rutile only or a mix of anatase and rutile, even if the XRD revealed really broad peaks with low intensity. In spite of a higher index and interesting features (compactness, crystallinity), samples M7 and M9 (group 2) are not an option toward photonic application due to grain growth at the sample surface. Indeed, crystallization may generate inhomogeneity such as grain boundaries inside the layer, which will affect the losses of photonic devices by diffusion at the interfaces or even disturb nanofabrication steps such as etching. The index of sample M5, which was also from the second group but with less advanced state of crystallinity, was close to 2.38 before annealing and up to 2.61 with a homogeneous but slightly porous morphology, which was fully compatible with the optical applications targeted. Results were also promising with the third group’s M14 sample, obtained by decreasing the deposition pressure to 14 mTorr, and the M10 sample, which combined high ICP power with moderate table power and pressure, with a refractive index close to 2.5 for their thick layer version prior to annealing, which can increase further after annealing at 600 °C. Both had a homogeneous morphology and, as previously underlined, they are expected to be partially crystalline at the nano-scale, so with no detrimental features for propagation compared to M7 or M9. We considered that we could produce, thanks to ICPCVD, at least three layers that are excellent candidates for the targeted photonic applications. We are currently adapting our lithography and etching processes for the waveguide fabrication in order to provide propagation loss results on those films.

## 5. Conclusions

We carried out an investigation on three dominating parameters for ICPCVD deposition (plasma source power, secondary plasma power, pressure) to simultaneously improve the refractive index and morphology of the TiO_2_ layers. To understand the trends drawn by this parametric study, we performed XRD analysis on each sample and related it to their measured refractive index; some variations of the index were weighted by the morphology and while not fully predictable, they will be of concern for future application.

We selected the five deposition conditions with the highest index to produce thick layers, suitable for propagation characterization after structuration in the form of waveguides. The increase in the deposition time and annealing confirmed that crystallization in both anatase and rutile occurred, even on the layers where the XRD spectra exhibited only non-defined peaks (or even just bumps) as-deposited, and TEM observations on one of the crystalline layers revealed that the crystallite was a few tens of nanometers wide, with no visible grain boundaries. Based on these observations, we determined three deposition conditions that are interesting to produce optical layers that will undergo a full nanofabrication process.

## Figures and Tables

**Table 1:** Values of deposition parameters and corresponding refractive index, bias value, comments on layer morphology (Col. = Columnar, Por. = Porous, C. = Compact, G. = Grains), and XRD pattern.

Sample	M1	M2	M3	M4	M8	M30	M5	M7	M9	M10	M11	M12	M14	M17
Table power (W)	0	100	50	50	50	50	50	50	100	50	100	50	50	50
Source power (W)	1000	1000	800	1200	1000	1000	1000	1500	1500	1500	1500	1000	1000	1000
Pressure (mTorr)	50	50	50	50	50	30	20	50	50	20	20	12	14	17
Average bias (V)	0	345	240	260	250	230	195	245	360	150	250	145	165	170
Refractive index @630 nm	2.00	2.17	2.16	2.17	2.13	2.20	2.33	2.28	2.30	2.33	2.19	2.28	2.28	2.32
Morphology	Col.	Por.	Col.	C.	C.	C.	Por.	C.G.	C.G.	Por.	C.	Por.	Por.	Por.
XRD pattern	Amorphous						One broad peak			Several broad peaks				

**Table 2:** Refractive index, film thickness, and figure of merit S obtained for the five chosen deposition conditions.

Sample	M14	M14T	M5	M5T	M10	M10T	M9	M9T	M7	M7T
Thickness	245	495	270	550	225	650	385	435	340	555
Refractive index	2.29	2.47	2.33	2.38	2.33	2.51	2.30	2.37	2.28	2.31
Figure of merit S	0.077		0.022		0.043		0.242		0.030	

## Data Availability

Data underlying the results presented in this paper are not publicly available at this time, but may be obtained from the authors upon reasonable request.

## Appendix A

We present typical XPS spectra obtained on M1, M2 M3, and M4 layers in Figure A1. The elements detected were mainly titanium and oxygen, as revealed by the high values for these two elements in the embedded quantification table, and carbon attributed to surface pollution (the carbon concentration was less than 3%at. in the depth of the layer during further investigations).
